# The association of PD-L1 expression and CD8-positive T cell infiltration rate with the pathological complete response after neoadjuvant treatment in HER2-positive breast cancer

**DOI:** 10.1007/s10549-023-07242-1

**Published:** 2024-01-25

**Authors:** Kenan Çetin, Şermin Kökten, Bahar Sarıkamış, Sedat Yıldırım, Oruç Numan Gökçe, Nagehan Özdemir Barışık, Ülkan Kılıç

**Affiliations:** 1 Department of General Surgery, Faculty of Medicine, Çanakkale Onsekiz Mart University, Çanakkale, Turkey; 2 Department of Pathology, University of Health Sciences, Kartal Dr. Lutfi Kırdar Training and Research Hospital, Istanbul, Turkey; 3 Department of Medical Biology, Faculty of Medicine, University of Health Sciences, Istanbul, Turkey; 4 Department of Medical Oncology, University of Health Sciences, Kartal Dr. Lutfi Kırdar Training and Research Hospital, Istanbul, Turkey

**Keywords:** HER2 + breast cancer, Neoadjuvant therapy, Response, CD8, PD-L1

## Abstract

**Purpose:**

Achieving a pathological complete response (pCR) after neoadjuvant therapy in HER2-positive breast cancer patients is the most significant prognostic indicator, suggesting a low risk of recurrence and a survival advantage. This study aims to investigate clinicopathological parameters that can predict the response to neoadjuvant treatment in HER2 + breast cancers and to explore the roles of tumour-infiltrating lymphocytes (TILs), CD8 + T lymphocytes and PD-L1 expression.

**Methods:**

This single-centre retrospective study was conducted with 85 HER2-positive breast cancer patients who underwent surgery after receiving neoadjuvant therapy between January 2017 and January 2020. Paraffin blocks from these patients were selected for immunohistochemical studies.

**Results:**

A complete pathological response to neoadjuvant treatment was determined in 39 (45.9%) patients. High Ki-67 index (> 30%), moderate to high TIL infiltration, PD-L1 positivity and high CD8 cell count (≥ 25) were significantly associated with pCR in univariate analyses (p: 0.023, 0.025, 0.017 and 0.003, respectively). Multivariate regression analysis identified high Ki-67 index (> 30%) and CD8 cell infiltration as independent predictors for pCR in HER2-positive breast cancer.

**Conclusions:**

High Ki-67 index, and high CD8 cell count are strong predictors for pCR in HER2-positive breast cancer. Tumours with high Ki-67 index, high TILs and CD8 infiltration may represent a subgroup where standard therapies are adequate. Conversely, those with low TILs and CD8 infiltration may identify a subgroup where use of novel strategies, including those that increase CD8 infiltration could be applied.

## Introduction

Globally, breast cancer is the most diagnosed malignancy, with over two million cases reported each year [1]. It is also a leading cause of cancer-related deaths among women worldwide. It is a heterogeneous disease group consisting of different subtypes that exhibit varying tumour biology and behaviour. Approximately 20–30% of all breast cancers overexpress the human epidermal growth factor receptor 2 (HER2) [2]. Breast cancers that overexpress HER2 can achieve a pathological complete response (pCR) with anti-HER2 agents when given in combination with chemotherapy. The pCR rates for these treatments range from 46 to 66% [3, 4]. The response to neoadjuvant chemotherapy has prognostic significance in patients with HER2-positive breast cancer. pCR after neoadjuvant treatment is the most important prognostic indicator, predicting a low risk of recurrence and providing a survival advantage [5]. Therefore, there is an ongoing effort to develop new treatment strategies aimed at increasing pCR rates and reducing the risk of recurrence in patients who do not achieve pCR. These strategies include combining trastuzumab and pertuzumab in neoadjuvant treatment and continuing adjuvant therapy with trastuzumab-emtansine (T-DM1) for patients who do not achieve pCR [6, 7]. However, there is still a subgroup of patients, approximately 25%, who show progression and have a poor prognosis despite neoadjuvant treatment [8]. Therefore, there is an urgent need to find accurate and reliable biomarkers that can predict who will benefit from this treatment.

In the treatment of HER2-positive breast cancer, achieving pCR has gained increasing value, in addition to being an important prognostic marker, for determining post-surgical adjuvant therapy. Therefore, understanding the role of tumour-infiltrating lymphocytes (TILs) and the immune response in achieving pCR is clinically important. Immune cells present in the tumour microenvironment are called TILs. These lymphocytes are believed to exhibit anti-tumoral effects in HER2-positive breast cancer through immune-mediated tumour-host interactions and antibody-dependent cellular cytotoxicity (ADCC), as observed in numerous other cancer types [9, 10]. Initially, studies have reported that patients with high TIL levels have shown a positive response to neoadjuvant treatment with trastuzumab, an anti-HER2 monoclonal antibody [11–13]. However, another study has reported that high levels of TILs are associated with treatment resistance [14]. As a result, the effects of TILs on neoadjuvant treatment in HER2-positive breast cancer patients are still not fully understood.

Programmed death-ligand 1 (PD-L1) is a transmembrane protein belonging to the B7 immune molecule family. It is present in both tumour cells and immune cells. It mediates tumour immunosuppression and is associated with immune evasion by tumour cells. Studies have shown that trastuzumab may influence the expression of PD-L1 in CD8 + T cells and cancer cells in HER2-positive breast cancer [15, 16]. It has been reported that trastuzumab can diminish the effects of PD-L1 on cancer cells by inhibiting HER2 [15]. Additionally, the PANACEA study proposed the hypothesis that trastuzumab can reverse tumour-mediated immunosuppression and activate local anti-tumour immune responses [17].

This study aims to investigate the clinicopathological parameters that can predict the response to neoadjuvant treatment in HER2 + breast cancers and to examine the role of the immune system by exploring the role of TILs, CD8 + T lymphocytes and PD-L1 expression in order to gain a better understanding of it.

## Materials and methods

### Study cohort

In our single-centre retrospective cohort study conducted at SBÜ Kartal Dr Lütfi Kırdar Health Practice and Research Centre, we included patients diagnosed with HER2-positive invasive ductal histopathological subtypes of breast cancer who underwent surgical intervention after neoadjuvant treatment between January 2017 and January 2020. The diagnosis was made through a core biopsy, and the HER2 status was determined using HER2 immunohistochemistry (IHC) 3 + or the HER2 gene amplified by fluorescence in situ hybridisation (FISH). HER2 IHC and HER2 FISH results were interpreted by expert breast pathologists (Ş.K. and N.Ö.B.) according to the 2018 ASCO/CAP recommendations [18]. The pathology file numbers and paraffin block numbers of the patients intended to be included in the study cohort (*n* = 98) were obtained from the archives of the pathology clinic. Paraffin blocks from all patients were examined, and those either with missing paraffin blocks or deemed unsuitable for immunohistochemical studies were excluded (*n* = 13). Eighty-five eligible patients were identified for inclusion; they underwent immunohistochemical studies to evaluate PD-L1 expression and CD8 + TIL count.

Physical examination, breast and axillary ultrasonography, mammography, ± breast MRI and PET-CT were used for staging patients. In cases where there was suspicion of axillary lymph node metastasis during physical examination and imaging, the metastatic status was evaluated using an ultrasound-guided fine-needle aspiration biopsy (FNAB) of the suspicious lymph node. In our clinic, patients with a tumour size > 2 cm in the breast and/or histopathologically confirmed axillary metastasis, as well as those with a suitable general health status, were considered candidates for neoadjuvant treatment.

All patients received neoadjuvant treatment targeting HER2 (trastuzumab ± pertuzumab) and chemotherapy. As part of the chemotherapy treatment, patients were treated with either a platinum-based or an anthracycline-based protocol. Pretreatment clinical and pathological variables were retrospectively evaluated from patient records. These variables included age at initial diagnosis, tumour size, lymph node status, hormone receptor status (defined as ≥ 1% nuclear staining of tumour cells for oestrogen and progesterone receptors), Ki-67 proliferation index (patients were categorised into two groups based on Ki-67 indices: low: ≤30%, high: >30%), histological grade (assessed using the Nottingham Histologic Score System), nuclear grade, multifocality and the specific neoadjuvant treatment received (trastuzumab, with or without pertuzumab). The assessment of tumour response was conducted on specimens obtained from excision and categorized into two groups: pCR and non-pCR. pCR was identified by the absence of invasive carcinoma and tumour thrombi in the lymphovascular channels within the breast, along with no evidence of metastasis in the axillary lymph nodes at the time of the surgical procedure (ypT0/ypTis and ypN0). Additionally, patients were categorized based on their response to neoadjuvant treatment in the breast, as either non-responsive (no definite response to presurgical therapy in the invasive carcinoma) or responsive (probable or definite response to presurgical therapy in the invasive carcinoma or no residual invasive carcinoma is present in the breast after presurgical therapy).

The study protocol was approved by the ethics committee of Dr. Lutfi Kırdar Kartal Research and Training Hospital, affiliated with the University of Health Sciences (reg. 2020/514/188/9).

### Immunohistochemical evaluations

Immunohistochemical stainings were performed on core biopsy specimens of the tumors, which were taken prior to neoadjuvant treatment, to establish the histopathological diagnosis of the disease. Immunohistochemical applications for PD-L1 and CD8 were conducted at the Department of Medical Biology, Hamidiye Medical Faculty, Health Sciences University (Ü.K. and B.S.). The evaluations were conducted by a specialized breast pathology research team, which was blinded to the clinical and pathological variables (Ş.K. and N.Ö.B.).

Histopathological evaluation of TILs was performed using H&E staining. All mononuclear cells including lymphocytes and plasma cells were included in the assessment, while granulocytes and other polymorphonuclear leukocytes were excluded. The TIL count was defined as the percentage of the stromal area adjacent to the tumour that is occupied by mononuclear cells (interposing between tumour nests). The expression of TİLs was categorised into three groups by modifying the International Working Group criteria [19]: low (TILs: 0–10%); moderate (TILs: 10–40%); high (TILs: 40–90%). Due to the limited number of patients with a high TIL ratio (n:10), we compared patients with moderate and high TIL ratios to those with a low TIL ratio.

**Fig. 1:**
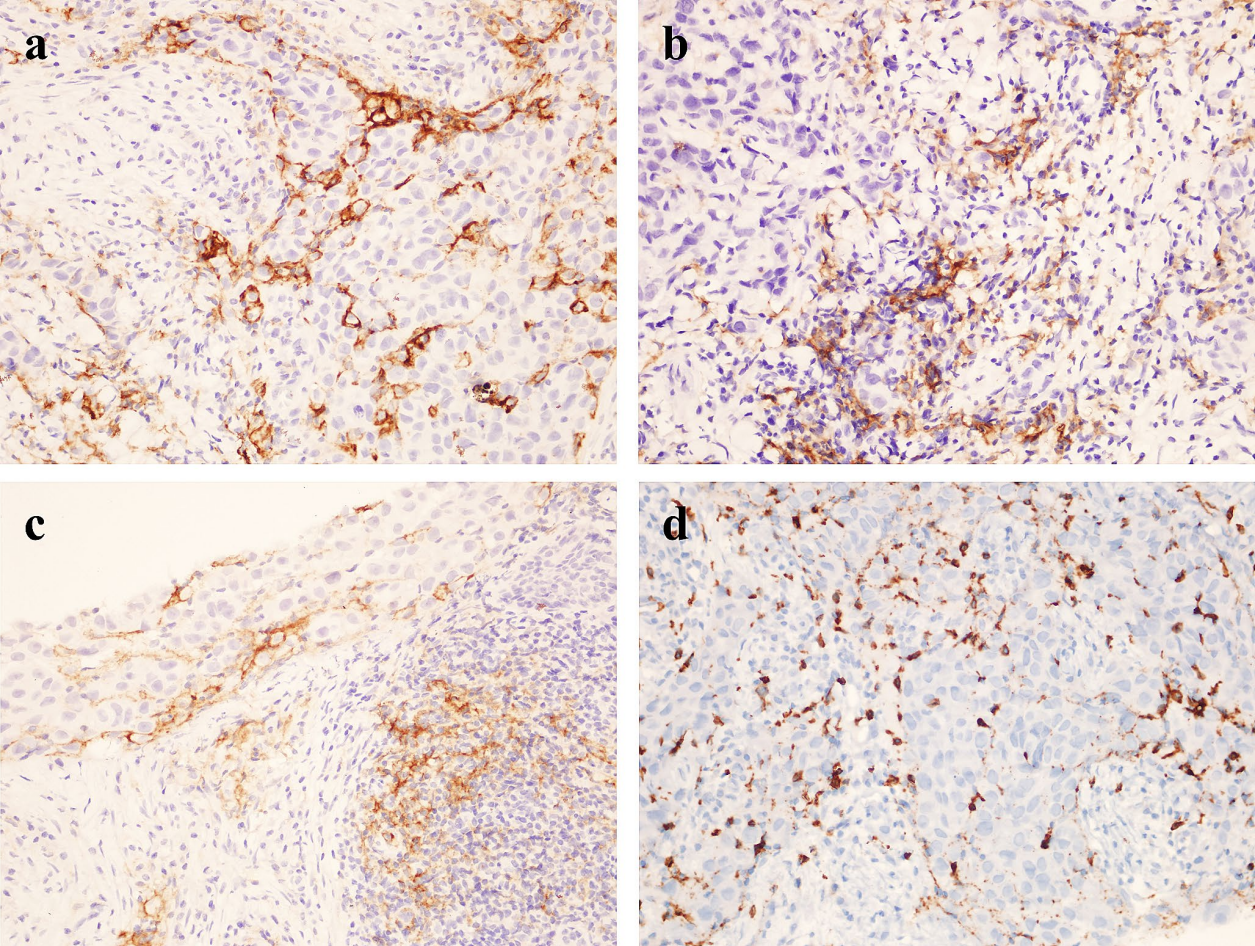
Microscopic immunostaining images of PD-L1 and CD8-positive lymphocytes in the stroma of breast cancer (X40); **a** PD-L1 in tumour cells, **b** PD-L1 in tumour-infiltrating lymphocytes, **c** PD-L1 in both tumour cells and lymphocytes, **d** CD8-positive lymphocytes infiltrating the tumour

Immunohistochemical analyses were performed using an autostainer (Leica Bond-III; Leica Biosystems, Bannockburn, IL, USA), following the manufacturer’s instructions. To detect PD-L1, a monoclonal antibody, 73–10 (Leica Biosystems, Newcastle, UK), was used. We evaluated PD-L1 expression on the TILs of all samples, assessing it as the proportion of the tumor area occupied by PD-L1-positive TILs of any intensity, following previously reported methods [20, 21]. The tumor area included viable tumor cells, associated intratumoral stroma, and contiguous peritumoral stroma. PD-L1 positivity was determined by the percentage of PD-L1-positive TILs relative to the total TIL count. A sample was defined as positive (PD-L1 + TILs) if PD-L1-expressing TILs comprised ≥ 1% of the tumor area [20, 21]. For PD-L1 expression on tumor cells (TCs), we assessed the proportion of viable invasive carcinoma cells showing any intensity of membranous staining, compared to the total number of such cells [21–24]. A positive PD-L1 expression on TCs was defined as ≥ 1% (PD-L1 + TC) [21–24] (Fig. 1a–c).

CD8 expression was assessed by IHC using the IR623 CD8 (C8/144B) RTU antibody (DAKO, Santa Clara, CA, USA). CD8 + T cells were counted within and around the tumour area (Fig. 1d). Based on the average absolute number of positively stained cells in 10 high-power fields (HPF), patients with < 25 cells were categorised as having low CD8 positivity, while those with ≥ 25 cells were categorised as having high CD8 positivity.

### Statistics and data analysis

SPSS version 24 (IBM Corp., Armonk, NY, USA) was used for all analyses. The normality of data distribution was assessed using the Kolmogorov–Smirnov test. Data were expressed as the mean ± standard deviation (SD) for continuous variables and as the median (min–max) for non-normally distributed quantitative variables. Qualitative variables were presented as counts and percentages. The independent samples t-test was used for comparing two groups of quantitative variables with normal distribution, while the Mann–Whitney U test was employed for those without normal distribution. Pearson’s Chi-square test and Fisher’s exact test were used for comparing qualitative data. Both univariate and multivariate logistic regression analyses were applied to identify variables strongly associated with pCR. A p-value of ≤ 0.05 was considered statistically significant.

## Results

### Study cohort

A total of 85 patients with HER2-positive breast cancer who underwent neoadjuvant treatment followed by surgery were included in the study. All patients were female, with a mean age of 49.5 ± 11.2 years (median: 50; range: 27–82). The mean tumour size was 30.5 ± 15.2 mm (median: 27 mm; range: 9–92 mm). The clinical and pathological characteristics of the 85 patients at the time of diagnosis are summarised in Table 1.

**Table 1:** Demographic and clinicopathological characteristics of the study cohort

Parameters	N (%) or mean ± SD or median/range
Age (year), mean ± sd	49.5 ± 11.2
*Menstrual status*	
Menopause/non	39 (45.9)/46 (54.1)
cT (mm), mean ± sd	30.5 ± 15.2
*cN*	
Negative/positive	7 (8.2)/78 (91.8)
*Tumor focality*	
Unifocal/multifocal	51 (60)/34 (40)
*Nucleus grade*	
Grade 2/3	45 (52.9)/40 (47.1)
*Histological grade*	
Grade 2/3	50 (58.8)/35 (41.2)
*ER status*	
Positive/negative	49 (57.6)/36 (42.4)
Rate (%), median/range	40/0-100
*PR status*	
Positive/negative	30 (35.3)/55 (64.7)
Rate (%), median/range	0/0–90
*Ki-67 index*	
Low (≤ 30)/high (> 30)	26 (30.6)/59 (69.4)
Rate (%), median/range	40/10–85
*Subgroup*	
HR-positive	49 (57.6)
HR-negative	36 (42.4)
TIL	
Low/moderate/high	44 (51.8)/31 (36.5)/10 (11.8)
Rate (%), median/range	10/1–80
*PD-L1*	
Positive/negative	36 (42.4)/49 (57.6)
PD-L1 (+) TC	29/(34.1)
Rate^a^ (%), median/range	0/0–20
PD-L1 (+) TILs	32/(37.6)
Rate^b^ (%), median/range	0/0–50
*CD8*	
Low (< 25)/High (> 25)	56 (65.9)/29 (34.1)
CD8/TIL-rate (%), median/range	35/10–45

cT, primary tumor; cN, lymph node involvement; ER, estrogen receptor; PR, progesterone receptor; HR, hormone receptor; TIL, tumor-infiltrating lymphocytes; PD-L1, programmed death-ligand 1; TC, tumor cells

^a^PD-L1 positivity rate in TC

^b^PD-L1 positivity rate in TIL

In the histopathological and immunohistochemical evaluations, we found that among the patients, 44 (51.8%) exhibited low TIL expression, 31 (36.5%) displayed moderate TILs, and 10 (11.8%) had high TIL expression. PDL-1 positivity was detected in 36 patients (42.4%). This expression was observed in tumor stroma cells in 29 patients (34.1%) and in infiltrating cells within the tumor in 32 patients (37.6%). Additionally, high CD8 cell counts (≥ 25) were observed in 29 patients (34.1%) among the TILs (Table 1).

Among our patients, 61 (71.8%) received neoadjuvant treatment with trastuzumab + CT [4AC; n: 58 / 4AC + 4T; n: 3], and 24 (28.2%) received trastuzumab + pertuzumab + CT [4AC + (4–6) T; n: 17 / 4AC; n: 4 / (5–7) T; n: 3]. After receiving neoadjuvant treatment, 42 (49.4%) patients opted for breast-conserving surgery, while the remaining 43 (50.6%) patients underwent mastectomy. The procedures performed in the axilla were as follows: only sentinel lymph node biopsy (SLNB) in 60 (80%) patients; direct axillary dissection (AD) in 5 (5.9%) patients; and SLNB followed by AD in 12 (14.1%) patients. When postoperative pathological evaluations were performed, it was observed that 70 (82.4%) patients responded positively to neoadjuvant treatment, while the remaining 15 (17.6%) patients did not respond. In addition, we identified a complete pathological response to neoadjuvant treatment in 39 (45.9%) patients. When evaluating patients for axillary lymph node involvement, 61 (71.8%) patients were reported as SLNB negative after neoadjuvant treatment, and no further intervention in the axilla was required (Table 2).

**Table 2:** Neoadjuvant therapy of the cohort, surgical procedure performed, and response status

Parameters	N (%)
*Neoadjuvant treatment*	
Trastuzumab + CT	61 (71.8)
Trastuzumab + Pertuzumab + CT	24 (28.2)
*Surgical procedure - Breast*	
BCS	42 (49.4)
Mastectomy	43 (50.6)
*Surgical procedure - Axilla*	
SLNB	68(80)
AD	5 (5.9)
AD followed by SLNB	12 (14.1)
*Neoadjuvant treatment response*	
No response	15 (17.6)
Responsive	70 (82.4)
pCR	39 (45.9)
*Post neoadjuvant axilla (ypN)*	
Negative	61 (71.8)
Positive	24 (28.2)
*Post neoadjuvant breast (ypT)*	
Negative (ypT0, ypTis)	42 (49.4)
Positive (ypT1/2)	43 (50.6)

CT, chemotherapy; BCS, breast-conserving surgery; SLNB, sentinel lymph node biopsy; AD, axillary dissection; pCR, complete pathological response

### The association of clinicopathological parameters with the response to neoadjuvant treatment

In our study cohort, the responsive group to neoadjuvant treatment exhibited statistically significant higher rates of moderate to high TIL ratio, PDL-1 positivity, high CD8 cell count (in 10HPF), and CD8 cell ratio (≥ 25) compared to the non-responsive group (p: 0.02, p: 0.002, p: 0.01, p: 0.027 and p: 0.001, respectively) (Table 3).

**Table 3:** The association of clinicopathological parameters with response to neoadjuvant therapy

Parameters	Responsive (n:70)	Not-responsive (n:15)	p-value	pCR (n:39)	Non-pCR (n:46)	p-value
Age (year), mean ± sd	49.2 ± 10.5	50.6 ± 14.5	0.67	47.6 ± 11.0	51.1 ± 11.2	0.15
cT (mm), mean ± sd	30.2 ± 15.9	31.7 ± 11.9	0.72	30.3 ± 16.3	30.6 ± 14.4	0.92
cN (+), n (%)	64 (91.4)	14 (93.3)	0.81	36 (92.3)	42 (91.3)	0.87
Multifocality, n (%)	29 (41.4)	5 (33.3)	0.56	15 (38.5)	19 (41.3)	0.79
Nuclear grade 3, n (%)	33 (47.1)	7 (46.7)	0.97	18 (46.2)	22 (47.8)	0.88
Histological grade 3, n (%)	28 (40)	7 (46.7)	0.63	16 (41)	19 (41.3)	0.98
ER (+), n (%)	40 (57.1)	9 (60)	0.84	24 (61.5)	25 (54.3)	0.50
ER-rates, median/range	37.5/0-100	60/0-100	0.60	50/0–95	35/0-100	0.58
PR (+), n (%)	25 (37.5)	5 (33.3)	0.86	11 (28.2)	19 (41.3)	0.21
PR-rates, median/range	0/0–90	0/0–90	0.96	0/0–90	0/0–90	0.06
HR- negative, n (%)	30 (42.9)	6 (40)	0.84	15 (38.5)	21 (45.7)	0.50
Ki67 (> 30%), n (%)	50 (71.4)	9 (60)	0.38	32 (82.1)	27 (58.7)	0.02
Ki67-rate, median/range	40/15–85	40/10–70	0.61	45/25–85	40/10–80	0.21
TIL-rate (%), median/range	15/2–80	8/1–60	0.10	20/2–80	8/1–70	0.048
TIL (moderate & high), n (%)	38 (54.3)	3 (20)	0.02	24 (61.5)	17 (37)	0.03
PD-L1 (+), n (%)	35 (50)	1 (6.7)	0.002	22 (56.4)	14 (30.4)	0.03
PD-L1 (+) TC, n (%)	29 (41.4)	0	0.002	21 (53.8)	8 (17.4)	0.001
Rate^a^ (%), median/range	0/0–20	0/0	0.005	0/0–20	0/0–3	0.017
PD-L1 (+) TILs, n (%)	31 (44.3)	1 (6.7)	0.007	19 (48.7)	13 (28.3)	0.07
Rate^b^ (%), median/range	0/0–50	0/0–5	0.004	5/0–40	0/0–50	0.13
CD8^c^, mean ± sd	309 ± 337	111 ± 86	0.027	402 ± 399	166 ± 165	< 0.001
Rate^d^ (%), median/range	35/10–45	35/25–40	0.60	35/10–40	35/25–45	0.45
CD8 high (≥ 25), n (%)	42 (60)	14 (93.3)	0.01	20 (51.3)	9 (19.6)	0.003
ypN0, n (%)	56 (80)	5 (33.3)	0.001	–	–	–

cT, primary tumor size; cN, lymph node involvement; ER, estrogen receptor; PR, progesterone receptor; HR, hormone receptor; TIL, tumor-infiltrating lymphocytes; PD-L1, programmed death-ligand 1; TC, tumor cells; CD8, CD8-positive T lymphocytes; ypN0, absence of invasive cancer in axillary nodes after neoadjuvant treatment

^a^PD-L1 positivity rates in TC

^b^PD-L1 positivity rates in TILs

^c^Number of cells counted in 10 HPF

^d^CD8/TIL rate

### The association of clinicopathological parameters with pCR

In the pCR group, univariate analyses, as shown in Tables 3 and 4, revealed significant associations with several factors; high Ki67 index, TIL-rate, moderate to high TIL infiltration, PDL-1 positivity, CD8 cell count (in 10HPF), and CD8 cell ratio (≥ 25).

**Table 4:** The comparison with univariate and multivariate analyses of clinicopathological parameters with pCR

Parameters	Univariate analyses			Multivariate analyses		
	OR	95% CI	p-value	OR	95% CI	p-value
Ki67, (> 30) vs. (≤ 30)	3.21	1.18–8.80	0.023	3.05	1.04–8.95	0.043
Ki67-rate	0.99	0.96–1.01	0.23	–	–	–
ER, (+) vs. (−)	1.34	0.56–3.20	0.50	–	–	–
ER-rate	1.00	0.99–1.01	0.81	–	–	–
PR, (+) vs. (−)	0.56	0.22–1.39	0.21	–	–	–
PR-rate	1.02	1.00–1.04	0.09	–	–	–
TIL-rate	0.97	0.96–1.00	0.027	0.98	0.94–1.02	0.28
TIL^a^	2.73	1.13–6.58	0.025	1.75	0.25–12.30	0.57
PD-L1, (+) vs. (−)	2.96	1.21–7.22	0.017	1.07	0.18–6.47	0.94
PD-L1 TC-rate	0.69	0.41–1.16	0.16	–	–	–
PD-L1 TIL-rate	1.00	0.96–1.04	0.94	–	–	–
CD 8^b^	4.33	1.65–11.32	0.003	4.22	1.02–17.43	0.047
CD8/TIL-rate	1.01	0.94–1.09	0.72	–	–	–

ER, estrogen receptor; PR, progesterone receptor; TIL, tumor-infiltrating lymphocytes; PD-L1, programmed death-ligand 1; CD8, CD8-positive T lymphocytes

^a^TIL: moderate to high vs. low

^b^CD 8: high (≥ 25) vs. low (< 25)

In the multivariate analysis, after controlling for other covariates, only high Ki-67 index (> 30%) and a high CD8 ratio (≥ 25) showed a statistically significant association with pCR (Table 4). Having a high Ki-67 index (%30) increases the likelihood of achieving a pCR in the breast by approximately 3-fold [OR (95% CI): 3.1 (1.04–8.95), p: 0.043]. Additionally, prior to neoadjuvant treatment, a high infiltration of CD8 cells (≥ 25) in the tumour microenvironment increases the likelihood of achieving a pCR in the breast by approximately 4-fold compared to patients with low CD8 infiltration [OR (95% CI): 4.2 (1.02–17.43), p: 0.047].

### The association of clinicopathological features with PD-L1 expression

We found that patients expressing PD-L1 had a higher histological grade (grade 3), Ki-67 rate, moderate to high TIL expression and a high number of CD8 + T cells compared to those without expression (p values: 0.002, 0.04, < 0.001 and < 0.001, respectively) (Table 5).

**Table 5:** The association of clinicopathological parameters with PD-L1 expression

Parameters	PD-L1-positive (n:36)	PD-L1-negative (n:49)	p-value
Age (year), mean ± sd	49.4 ± 10.2	49.5 ± 11	0.98
cT (mm), mean ± sd	30.7 ± 13.6	30.2 ± 16.4	0.89
cN (+), n (%)	34 (94.9)	44 (89.8)	0.69
Multifocality, n (%)	13 (36.1)	21 (42.9)	0.66
Nuclear grade (3), n (%)	21 (58.3)	19 (38.8)	0.08
Histological grade (3), n (%)	22(61.1)	13 (26.5)	0.002
ER (+), n (%)	19 (52.8)	30 (61.2)	0.51
ER-rate (%), median/range	30/0–100	40/0–100	0.51
PR (+), n (%)	11 (30.6)	19 (38.8)	0.50
PR-rate (%), median/range	0/0–90	0/0–90	0.36
Ki-67 (> 30), n (%)	27 (75)	32 (65.3)	0.48
Ki67-rate (%), median/range	50/20–85	40/10–70	0.04
HR-negative, n (%)	17 (47.2)	19 (38.8)	0.51
TIL (moderate & high), n (%)	34 (94.4)	7 (14.3)	0.000
TIL-rate (%), median/range	30/5–80	5/1–70	0.000
CD8 high (≥ 25), n (%)	26 (72.2)	3 (6.1)	0.000
CD8^a^, mean ± sd	495.8 ± 355.3	111.4 ± 140.9	0.000
CD8/TIL-rate (%), median/range	35/25–45	35/10–45	0.51

cT, primary tumor size; cN, lymph node involvement; ER, estrogen receptor; PR, progesterone receptor; HR, hormone receptor; TIL, tumor-infiltrating lymphocytes; PD-L1, programmed death-ligand 1; CD8, CD8-positive T lymphocytes; ypN0, absence of invasive cancer in axillary nodes after neoadjuvant treatment

^a^Number of cells counted in 10 HPF

## Discussion

The most important prognostic indicator predicting a low recurrence risk and survival advantage in breast cancer subgroups after neoadjuvant therapy is pCR [5]. Therefore, numerous studies have been conducted to investigate predictors of pCR following neoadjuvant treatment up to the present day. So far, apart from a few clinicopathological factors such as HER2 expression in breast cancer, an accurate and widely used biomarker has not been discovered. The HER2 oncogene can affect the therapeutic efficacy of trastuzumab by inducing PD-L1 expression, lymphocyte infiltration and activation in the tumour microenvironment. This suggests a potential link between TILs and PD-L1 and trastuzumab efficacy [14, 24–26]. Several studies have reported that TILs and PD-L1 have predictive value in patients with HER2-positive breast cancer. However, debates on this topic are still ongoing [27–29].

While many HER2 + breast cancers show favorable responses to neoadjuvant therapy, limited information exists in the literature regarding clinical or tumor-specific factors predicting this response. This study aims to elucidate the role of the tumor immune microenvironment in predicting the response to neoadjuvant therapy in HER2 + breast cancers. Our findings indicate that TILs, PD-L1 expression, and specific CD8 + lymphocyte subtypes are associated with the response to neoadjuvant therapy in primary breast tumors of breast cancer patients. Moreover, high Ki-67 levels (> 30%), moderate to high TIL infiltration, PD-L1 expression, and high CD8 cell expression (≥ 25) are associated with achieving a pathological complete response (pCR). We also identified high Ki-67 levels (> 30%) and elevated levels of CD8 + cells in the tumor microenvironment as independent predictors of pCR. Recent literature includes studies investigating TILs, PD-L1 expression, and CD8 + cell count in predicting pCR induced by neoadjuvant therapy in HER2 + breast cancer. However, these studies did not compare non-responders with responders in primary breast lesions. Therefore, our study, which encompasses partial responders in addition to those achieving pCR, represents the first comparison of non-responders and responders in this context.

High Ki67 expression has been reported to have a significant correlation with the response to neoadjuvant therapy in HER2 + breast cancer patients [30–32]. This relationship has been demonstrated not only in HER2 + breast cancer but also in other subtypes [33]. This finding aligns with the understanding that rapidly proliferating tumour cells with a high Ki-67 index are sensitive to chemotherapy. These factors serve as valuable predictors of response and can provide useful information for predicting which patients are likely to achieve a pCR and which patients are at a higher risk of residual disease, requiring more intensive adjuvant treatment.

High TIL ratios in the tumour microenvironment have been shown to have a better prognosis even in high-risk breast cancer subtypes, including triple-negative tumours [34–38]. In HER2 + breast cancer, however, high TIL ratios are relatively rare compared to triple-negative tumours (reported as 16% vs. 20%, respectively) [39]. While some studies have reported high TIL ratios using a cutoff value of 50% in HER2 + breast cancer, others have used a 40% threshold and reported rates of 18% [31, 32]. In our study, we used a cutoff value of 40% and found a high TIL ratio of 12% in our cohort. Although conflicting studies exist [31, 40, 41], Solinas et al.’s meta-analysis, which included five prospective randomised trials, established a positive correlation between high TIL ratios and increased pCR rates in HER2 + breast cancer patients [12]. While the high TIL ratio in our study cohort was relatively low, our findings indicate that a combined moderate to high TIL ratio was associated with pCR. Conversely, a low TIL ratio (< 10%) at baseline was associated with non-responsiveness to neoadjuvant therapy. However, the relationship between moderate to high TIL ratios and pCR was not confirmed in the multivariate regression analysis.

PD-1 is expressed on the surface of lymphocytes, and its ligand, PD-L1, is expressed not only on lymphocytes but also on cancer cells [42]. These two proteins belong to the immune checkpoint protein family, which renders T lymphocytes ineffective. PD-1/PD-L1 expression in the tumour microenvironment suppresses the immune response of cytotoxic T cells (CD8) against cancer cells and represents the resistance of tumour cells to anti-tumour immunity. The discovery of this mechanism led to the development of PD-1/PD-L1 inhibitors for various types of cancer [43]. Among solid tumours, the clinical significance of PD-L1 expression has been extensively studied in triple-negative breast cancer, and immunotherapeutic treatments with PD-L1 inhibitors have been introduced for this patient group. However, the role of PD-L1 expression in HER2 + breast cancer remains uncertain. In a cohort study conducted by Kurozumi et al., which involved 126 HER2 + patients, PD-L1 expression was detected in tumour cells in 17.5% of the patients. The detection was done using clone SP142 at a dilution of 1:50 from Spring Bioscience, USA [32]. PD-L1 positivity showed a significant correlation with high TIL and high CD8 + cell infiltration. They also discovered associations between PD-L1 expression, TILs and pCR rates. In another cohort study involving 216 patients with locally advanced HER2 + breast cancer, Hou et al. reported similar results [23]. They found that 18% of the patients expressed PD-L1 (clone SP263, rabbit, Ventana), which correlated with high levels of TIL and pCR rates. On the other hand, when using a different PD-L1 antibody (clone E1L3N, 1:200, Cell Signalling, Beverly, CA), Zhao et al. were unable to establish a significant correlation between a low staining rate (9.2%) and pCR rates [31]. In our study using the 73 − 10 clone, we found a higher rate of PD-L1 expression (42.4%) in the tumour stroma compared to what has been reported in the literature. The use of different PD-L1 antibodies in published studies likely explains the variation in epitope recognition and staining rates of PD-L1. Additionally, Yosikawa et al. analysed the PD-L1 immunohistochemical profiles of triple-negative breast cancers using the 73 − 10, SP142 and E1L3N clones and reported that the 73 − 10 test showed a higher positivity rate in immune and tumour cells [20]. While we found a univariate correlation between PD-L1 positivity and pCR, we did not observe a significant relationship in the multivariate analysis. Therefore, in our cohort, PD-L1 expression was not found to be an independent predictor of pCR in HER2 + breast cancer. Consistent with the findings of Kurozumi et al., our results suggest that PD-L1 expression in HER2 + breast cancers is associated with a high histological grade, as well as with TILs and CD8 + T cell counts [32].

To our knowledge, there are very few studies investigating the relationship between the ratio of CD8 + T cells in the tumour stroma and the achievement of pCR induced by neoadjuvant therapy in patients with HER2 + breast cancer. Kurozumi et al. considered a CD8 + cell count > 25 in their cohort of 126 HER2 + breast cancer patients who underwent neoadjuvant therapy as ‘high CD8 + TIL expression’ and reported a positive correlation with pCR [32]. However, in Zhao et al.’s study, which included CD8 immunohistochemical staining in 67 patients, they compared the number of CD8 + cells in 10 HPFs and did not find a significant relationship [31]. In our study, we calculated the average number of CD8 + cells in 10 HPFs and defined patients with ≥ 25 CD8 + cells as the ‘high CD8 + TIL expression group’. We discovered a significant correlation between the presence of high CD8 + TILs and pCR in both univariate and multivariate analyses.

A significant limitation of our study is its relatively small sample size. Due to the small sample size, statistical comparisons of HER2 + molecular subtypes (HR-/+ HER2+) could not be conducted. Additionally, although our cohort predominantly received single-target (trastuzumab) + chemotherapy, the inclusion of patients who received dual-target (trastuzumab + pertuzumab) + chemotherapy disrupted the homogeneity of neoadjuvant treatment. Larger, multicentre prospective studies with high patient volumes and homogeneous neoadjuvant treatments are needed to confirm our findings.

## Conclusion

In conclusion, our findings demonstrate that CD8 + T cells and high Ki-67 index (> 30%) are strong and independent predictors of pCR. In this context, pre-identifying CD8 + T lymphocytes, which are a subset within TILs, for predicting pCR in neoadjuvant therapy and developing strategies to enhance CD8 + responses in patients may prove beneficial for achieving pCR. On the other hand, although we observed a PD-L1 positivity rate of approximately 42.4% using the 73 − 10 PD-L1 antibody in HER2 + breast cancer, it was not a strong and independent predictor of pCR. Our results highlighted a relationship between PD-L1 expression in the tumour microenvironment and high histological grade, TILs and CD8 + T cell infiltration. Also, our study establishes only correlations between TILs, CD8 and pCR, not causation. Large, multicentre studies are required to validate our results among larger patient cohorts who have received consistent neoadjuvant treatments.
